# Disentangling the dynamical underpinnings of differences in SARS-CoV-2 pathology using within-host ecological models

**DOI:** 10.1371/journal.ppat.1009105

**Published:** 2020-12-11

**Authors:** C. Jessica E. Metcalf, Bryan T. Grenfell, Andrea L. Graham

**Affiliations:** 1 Department of Ecology and Evolutionary Biology, Princeton University, Princeton, New Jersey, United States of America; 2 Princeton School of Public and International Affairs, Princeton University, New Jersey, United States of America; University of Pittsburgh, UNITED STATES

## Abstract

Health outcomes following infection with Severe Acute Respiratory Syndrome Coronavirus 2 (SARS-CoV-2) are remarkably variable. The way the virus spreads inside hosts, and how this spread interacts with host immunity and physiology, is likely to determine variation in health outcomes. Decades of data and dynamical analyses of how other viruses spread and interact with host cells could shed light on SARS-CoV-2 within-host trajectories. We review how common axes of variation in within-host dynamics and emergent pathology (such as age and sex) might be combined with ecological principles to understand the case of SARS-CoV-2. We highlight pitfalls in application of existing theoretical frameworks relevant to the complexity of the within-host context and frame the discussion in terms of growing knowledge of the biology of SARS-CoV-2. Viewing health outcomes for SARS-CoV-2 through the lens of ecological models underscores the value of repeated measures on individuals, especially since many lines of evidence suggest important contingence on trajectory.

## Introduction

Infection with Severe Acute Respiratory Syndrome Coronavirus 2 (SARS-CoV-2) can yield strikingly different health outcomes. Some people experience few or no symptoms, others have devastating health consequences, from mortality to chronic afflictions. This combination is what makes this virus such a formidable public health challenge. Asymptomatic infection enables SARS-CoV-2 to spread widely, since people can transmit the infection without knowing it, while severe outcomes have yielded devastating death tolls and challenged health systems around the globe.

Evidence on potential risk factors for severe outcomes with SARS-CoV-2 is growing, so far encompassing age [1,2], sex [2,3], and comorbities like obesity [4]. However, seemingly similar people still experience very different health outcomes. The reasons for this remain largely mysterious, yet viral, immune and physiological dynamics within individuals are likely to play an important role. Data on the time-courses of viral load, induced immune cells, signaling responses, and effectors are accumulating [5–7], and the timing of immune responses is increasingly recognized as an important element of health outcomes. For example, the interferon signaling proteins can be protective early in infection, but pathological later [8].

Such time-dependence implies that accounting for the dynamics of within-host processes could provide a powerful lens for understanding health outcomes. A well-established literature spanning a range of pathogens provides an important foundation to build on. Pioneering work modeling the within-host dynamics of HIV, for example, provided estimates of the life span of productively infected cells that importantly informed design of treatment regimes [9]. Furthermore, within-host models have indicated important roles for target cell depletion as well as the timing and magnitude of induced immunity in shaping the peaks and troughs of density for pathogens from malaria [10] to influenza [11], with implications for vaccine design.

**Fig 1:**
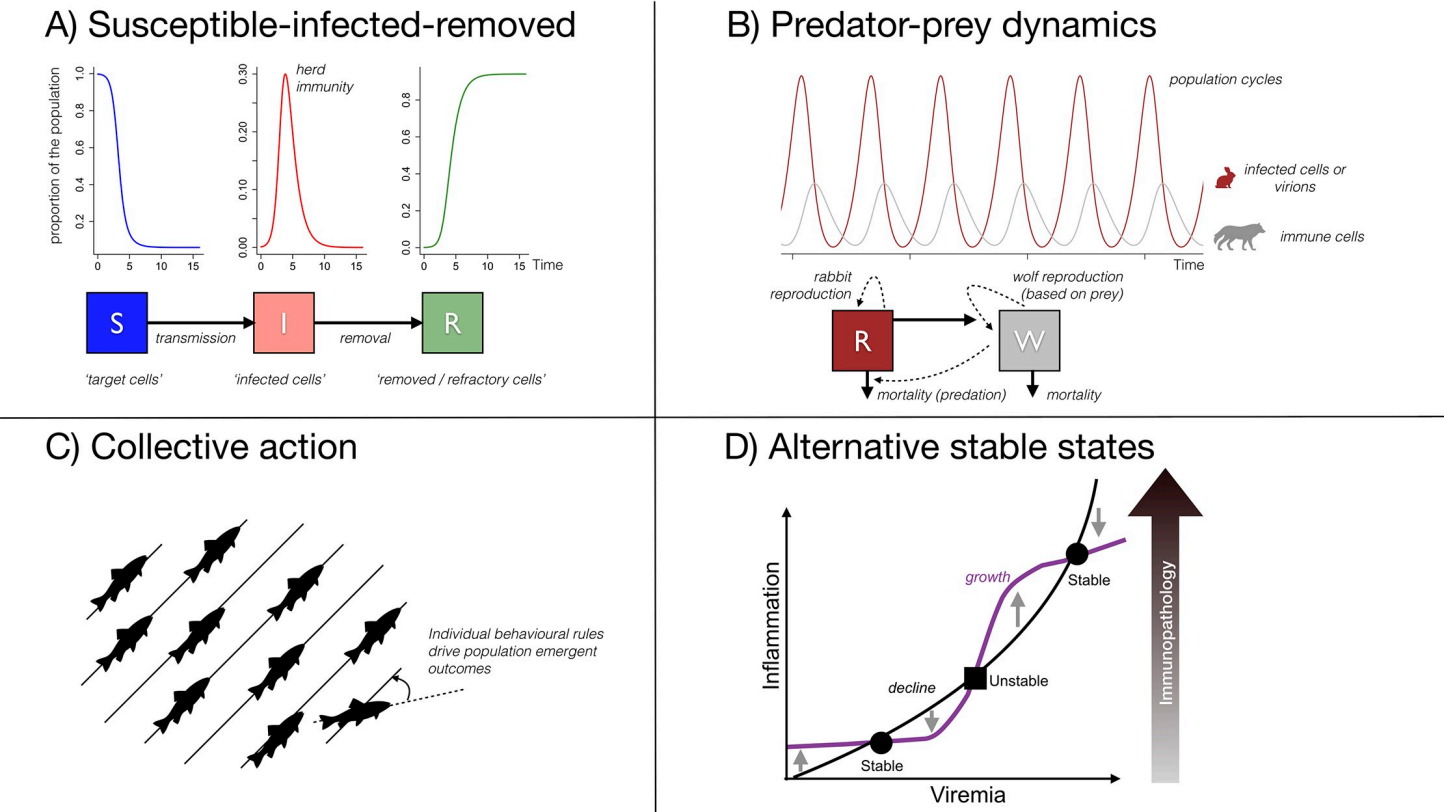
Ecological frameworks, and their translation toward within-host pathogen dynamics, including. (A) Susceptible-Infected-Removed models, a class of consumer resource models where individuals (or target cells) are initially susceptible (blue box/line), then may become infected (red) on exposure to an infected individual (or virion) via transmission (arrow); they are then removed (green box, line), e.g., via mortality. Susceptible depletion (declining blue line) eventually means that the proportion infected ceases to grow (herd immunity threshold). Early events (e.g., earlier virus detection via TLR7 in females) will have disproportionate effects on the trajectory of viral growth by compounding impacts on exponential growth. (B) Predator-prey models also broadly reflect consumer resource models, here with parameters illustrating population cycling (rabbit populations collapse as wolf populations (and thus predation) increase; but once rabbit numbers are too low to sustain wolf populations, these in turn collapse, and so on). Within-host, such compensatory dependence could explain similar peak viral loads (prey) across hosts (males and females) with different immune system features (predators), although repeated cycling as depicted here is likely to be rare. (C) In collective action models, simple rules at the individual level result in population level information integration (e.g., fish schooling). This might shape immune cell population coordination (e.g., CD4 T-cell fate selection), and disruption of this signaling could contribute to immunopathology; and (D) Alternative Stable States emerge if gradual changes push the state of the community (or immune system) to contexts that complicate return to initial conditions. Here, pro-inflammatory cytokines show accelerating and then staturating growth beyond a threshold of viremia (purple line abruptly increases then flattens) while anti-inflammatory cytokines grow consistently (black line curves smoothly upwards). The growth and decline in the inflammation are equal at the 3 points where the lines intersect. At 2 of these (circles), inflammation grows if it falls, and shrinks if it increases, indicating stable equilibria that may be hard to escape; the square represents an unstable tipping point. Small differences in the timing and magnitude of viremia/the inflammatory response might push some individuals above this tipping point while others remain below, driving very divergent health outcomes in otherwise similar individuals.

The duel between viruses and immunity manifests as a complex series of population interactions between cells, viruses, and signaling molecules—essentially a within-host ecological interaction. Here, we provide an overview of the potential as well as the limitations of ecological principles (Fig 1) to understand the within-host spread of the virus and the unfolding immune response. We discuss how, considered in this way, differences in immunity for which we have some understanding (e.g., due to age and sex), could help explain differential disease trajectories that remain mysterious for SARS-CoV-2.

### Susceptible-infected-recovered models

Susceptible-infected-recovered models (Fig 1A) describe the spread of infection between individuals. Parameters include R_0_, or the number of new infections per infected individual in a completely susceptible population, and the serial interval, or average time separating 1 infected individual from the next, which together define early spread. For example, with R_0_~2 and a serial interval of approximately 1 week, the number of infections doubles every week, aligning with explosive early growth of SARS-CoV-2 following introduction into communities around the world.

These principles can also describe spread of a virus like SARS-CoV-2 within hosts: Target cells in the lung (or other organs) that express the angiotensin-converting enzyme 2 (ACE2) receptor required for viral cell entry become the “susceptible” individuals. Leveraging known aspects of viral biology (e.g., in vitro replication indicates that the eclipse period, or interlude separating cell invasion to virus production, is approximately 6 to 8 hours), trajectories of viral load can be used to estimate R_0_ and the serial interval for within-host spread (S1 Text). To date, such approaches place the within-host R_0_ of SARS-CoV-2 between 3 and 8 [12,13], and potentially as high as 20 in the lower respiratory tract where this has been estimated separately [14]. Assuming a serial interval of less than a day, this indicates very rapid growth of the number of infected cells—more than tripling every day.

The magnitude of R_0_ also provides an approximation for the threshold for “herd immunity,” or the proportion of the susceptible pool that must be removed for the incidence of infection to start declining (S1 Text). Within-host, target cells can be removed from the susceptible pool by direct viral damage to the epithelial cells of the respiratory tract, or “friendly fire” from the immune system, as described for influenza [15]. Striking lung damage observed even in asymptomatic, SARS-CoV-2–positive individuals [16] suggests that loss of target cells could be sufficient to slow (although perhaps not stop) within-host viral spread. Uninfected cells can also be “removed” from the susceptible pool by the effects of the type 1 interferon pathway, a fast-acting early signaling cascade associated with innate immunity that causes cells to become refractory to infection [17,18]. In influenza in ponies, more than half the susceptible cells are estimated to be able to become refractory within 2 to 3 days [11]; importantly, this immune pathway seems to be often impaired by SARS-CoV-2 [19]. For a within-host R_0_ between 3 and 8, between 60% and 80% of the uninfected target cells would have to be resistant or otherwise made refractory for viral loads to start to decline (by analogy with the threshold for herd immunity) because 1 infected cell results in less than 1 new infected cell assuming that target cell availability is the key driver of viral spread. However, models also suggest that SARS-CoV-2 disseminates slowly through different regions of the lungs (driving the long duration of infection in some individuals) [14], so that relatively spatially restricted depletion of target cells to 60% or 80% could effectively slow or prevent spread.

**Fig 2:**
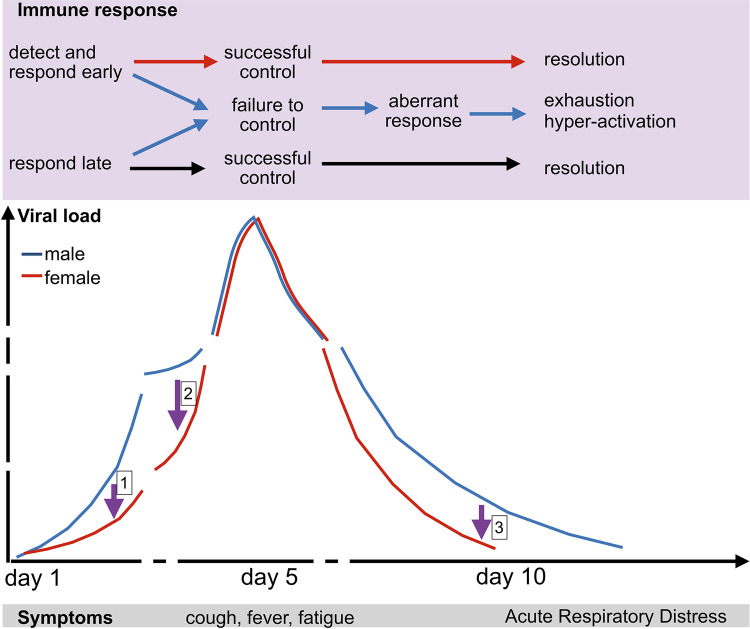
**Dynamics of SARS-CoV-2** showing the hypothesized trajectory of viral load in the respiratory tract (bottom panel) for males (blue) and females (red). The top panel broadly maps a set of potential immune responses, roughly corresponding to recently described immunotypes [5]: Detecting and responding early (e.g., via early activation of type I Interferons) leading to early resolution, a delayed but ultimately successful response (e.g., via moderate Type 1 T cell activation), or an aberrant response resulting in hyperactivation (e.g., cytokine storms and exhaustion of lymphocytes) and the most severe forms of disease. Hosts of different sexes or ages might differ in propensity to follow the possible trajectories suggested in the top panel. For example, strong early detection and response in females or younger individuals (purple arrow labeled 1) could result in lower early viral loads. Despite delayed control, males or older individuals might still be able to regain the lost ground by successful development of cellular immunity (purple arrow labeled 2); if this response is greater for greater viral loads, this could ultimately result in similar scales of viral load in both slow and fast responding individuals around the peak of viral load. Finally, exhaustion/hyperactivation (of adaptive and innate arms of the immune system, respectively), potentially shaped by events early during infection (“path dependence,” such as failure of early interferon defenses or by comorbidities), could result in slower clearance in males or older individuals (purple arrow labeled 3 would correspond to females/younger individuals).

Even if target cell depletion alone might not be of sufficient magnitude to stop the increase in viral load, such local effects are likely to shape the within-host dynamics in SARS-CoV-2—especially as anything that reduces early spread will have disproportionate effects in the context of exponential growth (in Fig 2, early reductions in viral growth indicated by the purple arrow labeled 1 result in rapid and substantial reductions in viral load as illustrated by the deviation between the red and blue lines). However, the start of the decline in viral load is likely to be largely driven by later-acting but more precisely targeted immune defenses, such as virus-specific cytotoxic T cells (potentially a correlate of protection for SARS-CoV-2, as noted for other severe coronaviruses [20]), rather than target cell depletion alone.

Known physiological sex differences generate predictions as to the early within-host growth of viral populations. Androgens increase expression of two cell receptors necessary for cell invasion by the virus, ACE2 [21] and transmembrane protease, serine 2 (TMPRSS2) [22]. Associated increased rates of cell invasion (*β* in S1 Text) could accelerate early viral population growth in males (Fig 2). Moving onto immunity, a critical part of the immune response is detection of the virus and triggering of appropriate signaling cascades. Since the virus seems particularly adept at disrupting interferon signaling [19,23], which provides protection by inducing cells to become refractory, any sex differences in this process might shape the trajectory of viremia. The X chromosome encoded pattern recognition receptor Toll-Like-Receptor 7 acts upstream of type 1 interferons and escapes silencing to some degree in females [24], thus potentially contributing to reduced burdens repeatedly observed in females in this pandemic [3]; evidence for worse SARS-CoV-2 infection outcomes for males with TLR7 mutations [25] further underscores the protective effect of this receptor (unfortunately, viral loads were not available for these patients). Immune effectors launched following pathogen detection may also show sex or age differences, with female neutrophils more responsive to type 1 interferon [26], again potentially leading to earlier reductions with potentially long-term implications predicted by the Susceptible-Infected-Recovered framework. Conversely, natural killer cells may be more abundant and more active in younger males (although the pattern reverses with age) [27]. At later ages, T cells may show diminished effectiveness in detecting and responding to infection [28] that may result in faster early growth leading to potentially worse outcomes.

Susceptible-infected-recovered models provide metrics for early within-host viral growth and identify the boundaries of viral growth, but leave open the details of how the immune response might be affected by, and affect these dynamics. Predator-prey models provide one direction to address this.

### Predator-prey models

Over the 5 days that, on average, separate SARS-CoV-2 infection from peak viral load (Fig 2), innate immune responses intensify and adaptive immune responses are recruited. The unfolding within-host dynamic can be readily conceptualized in terms of predators (immune cells) and prey (viral particles or viral infected cells). The metaphor of (specialist) predator-prey dynamics (Fig 1B) is inaccurate at a basic level: The survival of the predators is not directly contingent on consuming the prey, and “affinity maturation” in B cells allows predators to become more efficient at capturing prey over short time-scales. Yet, such coupling of “predator” (immunity) abundance to “prey” (virus) abundance could benefit the host: If immune cells are recruited and expanded only in the presence of the pathogen, fewer resources might be wasted, and the design required is relatively trivial. For example, patterns of T-cell expansion in response to antigen concentration suggests relatively simple competition dynamics dependent on antigen concentration [29]; recruitment might similarly be associated with antigen abundance [30].

Such effects may underpin one puzzling observation for SARS-CoV-2: Processes that should reduce early growth of the virus in some individuals (e.g., females with greater interferon responsiveness, etc.) tend not to map to an expected reduction in peak viral load. Indeed, peak viral load seems relatively similar across groups [31,32] and shows no clear relationship with severity [33,34] (although more severe cases may shed for longer [35], see below). Compensatory growth in immune effectors might drive this convergence to the same peak, by analogy with the “paradox of enrichment” principle from ecology. For example, a reported correlation between natural killer cells and viral load in SARS-CoV-2 [33] could lead to individuals with early high viral loads (e.g., males) having concomitant growth in immune effectors like natural killer cells that reduce viral load—and this could bring viral load in males in parity with females by the time of the peak. However, a threshold for “herd immunity” (where target cells define a hard ceiling on viral load, see above), as well as measurement uncertainty cannot be ruled out.

In general, individual level heterogeneity (whether due to sex, age, or other factors) can affect the course of predator-prey–like interactions for SARS-CoV-2 in a variety of ways. For innate immunity, neutrophil recruitment is more efficient in females (as mentioned above [26]), and males also seem to recruit a slightly different class of “predators,” with the proportion of nonclassical monocytes amplifying in response to concentration of the chemotactic CCL5 cytokine, a relationship not seen in females [6]. Adaptive immunity is generally qualitatively different in males and females, e.g., with more B cells, capable of recognizing a broader array of antigens in females, but a higher ratio of cytotoxic T cells in males [24]. In SARS-CoV-2, a limited T-cell response in males has been associated with worse outcomes [6], and this lacunae is amplified by age, which generally has the effect of diminishing effectiveness of adaptive immune cells [36]. Conversely, while some predators are “protective,” others may not be: High antibody titres later (associated with B cells) can be associated with worse outcomes in females [37], and the simplest predator prey framing does not address the impacts of the multiple effectors of immunity, and their interactions.

Overall, the predator-prey metaphor may usefully address feedback between immune activation and viral load, capturing the fact that as pathogen numbers grow, so too might the population of the agents of their control. However, this framing where “predators” respond independently to “prey” density equates to simplifying the diverse and highly integrated set of immune effectors down to a single entity (the “predator”) and thus does not capture the complex coordination that occurs across the immune system and is central to its effectiveness.

### Collective action

For SARS-CoV-2, in addition to antigen-driven activation of virus-specific CD8 T cells, molecular signatures associated with the robust T-cell response in hospitalized patients point to bystander activation and homeostatic proliferation [38]. This wide-ranging set of triggers of proliferation of T cells is potentially in part an adaptation to prevent hijacking of the immune system [39], but is also likely to reflect adaptation to integrate information and coordinate activity across the diversity of players in the immune system. Collective action models (Fig 1C) speak to this [40,41]. Simple rule sets at the level of individuals (here, immune cells) can result in high-level emergent properties that reflect critical information processing at the level of the group (here, populations of immune cells). Increasing evidence of unexpected disjunctions in the sets of immune cell communities detected in patients with the most severe cases suggest that one of the reasons that SARS-CoV-2 pathology emerges is because this virus is somehow disrupting mechanisms underpinning coordinated behavior [38], with bad outcomes associated with, for example, T-cell-independent B cell responses. Signatures of such disruption might be evoked by reversal of expected sex differences, with, for example, greater antibody responses in males for SARS-CoV-2 [42], which is at odds with data from a wide range of pathogens and vaccinations [24]. However, attributing this specifically to disruption of signaling associated with collective action is not straightward. It remains relatively early days for considering how such models might be relevant to immune function, let alone applied insights from this.

A final potentially useful ecological framing emerges from the fact that disruption of immune function (either in terms of collective action, or more generally) could result in failure to control the virus, but can also risk unleashing life-threatening inflammation, especially when placed in the context of regulatory feedbacks that raise the possibility of alternative stable states.

### Alternative stable states

A surprising feature of SARS-CoV-2 is that the worst syndromic outcomes (e.g., Acute Respiratory Distress) occur after viral loads have reached low levels. The feedback loops that govern the immune system in tandem with the impact of “friendly fire” might drive this, a phenomenon most famously manifested in “cytokine storms.” Pinning down how this happens with precision is complicated by the fact that “cytokine storm” has no clear quantitative definition [43]. Although elevated levels of the cytokine interleukin 6 (IL-6) are often identified as a key correlate, SARS-CoV-2 levels of IL-6 generally fall far short of those noted in influenza cytokine storms [43]. This may be because IL-6 is a correlate rather than a driver of the associated inflammation, but it might also reflect the more nuanced issue that a “cytokine storm” is likely to denote a path-dependent outcome, i.e., one that depends not just on the state of a system, but also the history of how the system got to that state, and thus is hard to measure using a single quantity like IL-6 at a single time point.

Such path dependence is addressed by another ecological framework, i.e., that of alternative stable states (Fig 1D). Feedbacks inherent in ecological (and immuno-) dynamics can lead to tipping points that separate distinct equilibria that can only be reached or escaped when a driving variable (hunting [44] or fire frequency [45]) follows particular trajectories. Different branches of immunological signaling promote or suppress inflammation, often around specific equilibria, also referred to as set points [46]. If such set points are also context dependent (e.g., if the equilibrium degree of inflammation depends on viral load), alternative stable states [47,48] may emerge (Fig 1D shows 1 possible conformation). The role of the ACE2 receptor not only in virus spread but also in dampening inflammation may create particular vulnerabilities to this outcome (also noted for high pathology influenza [49]).

Various signs point to such path dependence. For SARS-CoV-2, interferon is protective early in disease but later becomes pathogenic [8], perhaps partly as it may also be up-regulating ACE2 in airway epithelia [50]. The schematic in Fig 1D suggests that this could arise if early in the infection, interferon is helping the immune system reach a first protective equilibrium, where inflammation drives down viral incidence without causing too much damage, and low viral load then mutes subsequent immunological activity; whereas later, interferons are forcing the system to stay at the second problematic equilibrium associated with significant levels of immunopathology. This might also help explain the fact that B and T-cell populations remain elevated an entire week in severe cases of SARS-CoV-2 [38], by contrast with the few days that tend to follow other viral infections or vaccination; the immune system has been caught in a problematic and self-reinforcing stable state and remains there.

The framing of alternative stable states often hinges on stochastic forcing, where relatively small chance events might push individuals from one peak to another [44]. Such small differences either in viral load or in the individual inflammatory context (*x* and *y* axes, Fig 1D) might drive the extraordinary variety in health outcomes observed in SARS-CoV-2. As individuals age, levels of inflammation also tend to increase [51] shifting individuals up the *y* axis on the inset on Fig 1D; and worse outcomes for Coronavirus Disease 2019 (COVID-19) might therefore be more frequent. Higher innate immune cytokines led to worse outcomes relative to healthy volunteers in females over males [6], in line with more active female immune systems. Conversely, overall, viral loads seem pretty similar between the two sexes [31] and between symptomatic and asymptomatic infections [52], although symptomatic males might have slightly higher loads than asymptomatic males [53], aligning with the possibility that male symptoms are associated with a failure to control the infection, that might tip them over into a stable state associated with worse health outcomes. Duration of viral shedding might also be longer in males [37,54] and associated with worse health outcomes [35], potentially also by allowing the transition to an alternative stable state.

Beyond the acute and extreme symptoms often bracketed under the heading “cytokine storm,” even mild SARS-CoV-2 infections may be followed by persistent symptoms reminiscent of chronic fatigue syndrome or myalgic encephalomyelitis (“long-haulers”). Such patterns are also suggestive of having reached a problematic immune and metabolic equilibrium that is hard to reverse, and might be better understood by considering individual trajectories of immunity and physiology.

## Discussion

Variation, but also consistency, in measures of SARS-CoV-2 infections has been notable. For example, peak viral load shows little variation by sex or by age, despite these factors being associated with known and profound differences in immune system functioning. As we outline above, a dynamical perspective suggests candidate ecological feedbacks that could explain this: For instance, target cell depletion might reduce viral spread at similar incidence (analogous to the concept of herd immunity); self-limiting predator-prey dynamics dictated by interactions with immune cells might have a similar effect.

An ecological perspective also points to measurements that might illuminate as yet undetected drivers of variation in health outcomes. Alternative stable states will only emerge in the context of relatively specific relationships linking driving variables and immunological activation (Fig 1D). Slight changes in approaches to plotting and analyzing the increasingly rich body of longitudinal data on immune measures available [5,6,38] could be used to identify whether such patterns emerge, e.g., laying trajectories out as illustrated on Fig 1D, and quantifying slopes and nonlinearities. Further, a key feature of alternative stable states may be path dependence—i.e., the history of states matters as much as the current state—underscoring the value of longitudinal data, or repeated measures on an individual, in teasing apart drivers of pathology. Considering potential ecological drivers also illustrates ways in which drivers of variation might be elusive. If much of the surprising deregulation and lack of coordination across adaptive immunity recorded [38] is rooted in disruptions to features driving collective behavior, measurement is likely to be very challenging. Collective behavior will rely on transient interactions and plasticity at the individual cell level that may prove very hard to measure.

A better understanding of the within-host dynamics of infection could shape design and application (e.g., timing) for therapeutics from antivirals [12,13] to anti-inflammatories and also has potential to inform vaccine design. Placing these refinements within the broader population context, models could also be adapted to calibrate the role of protection associated with previous exposure to coronaviruses, via cross-reactive antibodies or T cells [55], of relevance for vaccination and its consequence. Our focus here has been on ecological modeling, but an evolutionary ecology perspective will grow in importance as deployment of vaccines generates important selection pressures on the pathogen. The apparently weak association between symptoms and transmission for SARS-CoV-2 suggests that virulence evolution in the wake of selection via vaccination [56] is relatively unlikely, but data-informed models may help bound the range of possible outcomes.

It is also clear that all four framings miss the mark in important ways, lacking in particular the full complexity of feedbacks in the immune system. An analysis focused on the tractable feedbacks (e.g., target cell depletion and viral load–dependent recruitment of natural killer cells) may obscure the role of the harder to measure and perhaps undefined feedbacks. Importantly, these dynamical processes might all effectively cancel out, such that the most practical approach to probing the immune context to better understand pathology in SARS-CoV-2 and project treatment strategies is simply to identify early cytokine profiles that have been largely shown to dictate disease progression [5] and use these to guide delivery of, e.g., interferons or catch later progression into immunopathology to deliver immunosuppressants [57].

To conclude, important dynamical features of any within-host infection include the early phase of exponential growth, control and decline of the infection (shaped by immune effectors and/or target cell depletion), and other complexities that emerge from nonlinear feedbacks, such as alternative stable states. Addressing each of these with ecological models has the potential to reveal system-level mechanisms of COVID-19 pathology, with potential applications from therapeutics to vaccination, once our understanding of (and measurement of) the molecular and cellular mechanisms are further enriched.

## Supporting information

S1 Text Estimating R0 at the within- and between-host scale and the concept of herd immunity. (DOCX)
