# Supplementary material for: Disentangling the dynamical underpinnings of differences in SARS-CoV-2 pathology using within-host ecological models
Source: PLoS Pathog. 2020 Dec 11;16(12):e1009105. doi: 10.1371/journal.ppat.1009105 (PMC7732095; doi:10.1371/journal.ppat.1009105)
Supplement: S1 Text — (DOCX) [file ppat.1009105.s001.docx]

**Supplement S1:** Estimating R_0_ at the within- and between- host scale, and the concept of herd immunity.

At the between-host scale, R_0_, or the number of new infections per infected individual in a completely susceptible population is defined by:

$$R_{0}=\frac{\beta}{\gamma}$$

where $\beta$ is the rate of transmission (shown by the arrow linking the ‘S’ and ‘I’ boxes on Fig 1A) and encompasses both the rate of encounter between susceptible and infected individuals and the probability of transmission on that encounter; and $\gamma$is the rate of recovery or death following infection (shown by the arrow linking the ‘I’ and ‘R’ boxes on Fig 1A). Herd immunity is defined as the threshold whereupon one infected individual infects less than one other individual, i.e., $R_{0}S<1$ where ‘S’ reflects the proportion of the population that is susceptible. If $R_{0}=2$, the number of infections will start to decline once half the population has been infected, or removed (e.g., by vaccination) i.e., $S=0.5$.

By analogy, at the within-host scale, we can write:

$$R_{0}=\frac{p\beta T_{0}}{\delta(c+\beta T_{0})}$$

where *T_0_* is the number of susceptible or target cells at the start of the infection, which may become infected by virions at rate $\beta$, *p* is the rate of virion release from infected cells, and *c* is the rate at which virions are cleared from circulation (e.g., by immune effectors such as antibodies), and $\delta$ is the rate at which infected cells are cleared [1]. With estimates of $R_{0}$between 3 and 8, the proportion of the susceptible population that must be removed for the number of infected cells to decline is at least 65%.

**References**

1 Gonçalves A, Bertrand J, Ke R, Comets E, de Lamballerie X, Malvy D, et al. Timing of antiviral treatment initiation is critical to reduce SARS-CoV-2 viral load. CPT Pharmacometrics *Syst Pharmacol*. 2020. doi:10.1002/psp4.12543
